# Cost-Effectiveness of an Opportunistic Screening Programme and Brief Intervention for Excessive Alcohol Use in Primary Care

**DOI:** 10.1371/journal.pone.0005696

**Published:** 2009-05-27

**Authors:** Luqman Tariq, Matthijs van den Berg, Rudolf T. Hoogenveen, Pieter H. M. van Baal

**Affiliations:** 1 Centre for Prevention and Health Services Research, National Institute of Public Health and the Environment, Bilthoven, The Netherlands; 2 Centre for Public Health Forecasting, National Institute of Public Health and the Environment, Bilthoven, The Netherlands; Aga Khan University Karachi, Pakistan

## Abstract

**Background:**

Effective prevention of excessive alcohol use has the potential to reduce the public burden of disease considerably. We investigated the cost-effectiveness of Screening and Brief Intervention (SBI) for excessive alcohol use in primary care in the Netherlands, which is targeted at early detection and treatment of ‘at-risk’ drinkers.

**Methodology and Results:**

We compared a SBI scenario (opportunistic screening and brief intervention for ‘at-risk’ drinkers) in general practices with the current practice scenario (no SBI) in the Netherlands. We used the RIVM Chronic Disease Model (CDM) to extrapolate from decreased alcohol consumption to effects on health care costs and Quality Adjusted Life Years (QALYs) gained. Probabilistic sensitivity analysis was employed to study the effect of uncertainty in the model parameters. In total, 56,000 QALYs were gained at an additional cost of €298,000,000 due to providing alcohol SBI in the target population, resulting in a cost-effectiveness ratio of €5,400 per QALY gained.

**Conclusion:**

Prevention of excessive alcohol use by implementing SBI for excessive alcohol use in primary care settings appears to be cost-effective.

## Introduction

Excessive alcohol use is a cause of morbidity and even mortality, as it increases risks of coronary heart disease, stroke, and several types of cancers, with associated losses of life-years and quality of life [1], [2], [3]. In addition, substantial disability from medical and psychiatric consequences, injuries and “secondhand” effects (e.g. motor vehicle crashes) are attributed to excessive use of alcohol [4].

In the Netherlands, about 1% of total mortality, 4.5% of the public burden of disease and 0.6% of total health care costs (in 2003) can be attributed to chronic diseases caused by excessive alcohol consumption [5]. Currently, about 14% of Dutch men aged 12 or above drink more than three alcoholic consumptions per day and about 10% of the Dutch women aged 12 or above drink more than two alcoholic consumptions per day [6].

As a result, effective prevention of excessive alcohol use has the potential to reduce the burden of disease in the Netherlands considerably. Brief intervention for excessive alcohol use in primary care settings is an effective intervention [7], [8], [9], [10], [11]. In a randomized study, Fleming et al. [7], [8] found significant reductions in 7-day alcohol use in patients who received brief physician advice 12 months after the intervention. Senft et al. [9] also found significant reductions in numbers of weekly drinking days 12 months after receiving the brief intervention. Bertholet et al. [10] indicated that brief intervention in primary care resulted in a reduction in weekly ethanol intake of 38 grams per person. Also randomized trials conducted in other settings have demonstrated a reduction in the intake of alcohol by three to nine drinks per week, as compared to the control group [11]. In order to recognize and treat patients with alcohol problems, an opportunistic screening program should be included in the general practices. Fiellin et al. [12] evaluated the accuracy of screening methods for alcohol problems in primary care. The Alcohol Use Disorders Identification Test (AUDIT) was most effective in identifying subjects with at-risk, hazardous, or harmful drinking. Sensitivity of this questionnaire ranged from 51% to 97%, while specificity ranged from 78% to 96%. While screening would be opportunistic and not targeted at any group specifically, brief intervention is targeted at groups with a high risk to be(come) an excessive drinker in primary care. High risk groups are defined as women who drink 2 or more standard alcohol drinks (i.e. >20 grams ethanol) per day; and men who drink 4 or more standard alcohol drinks (i.e. >40 grams ethanol) per day; without meeting the DSM-IV criteria for alcohol dependency [13].

In the present study, we performed a cost-effectiveness analysis (CEA) of SBI in primary care patients. The outcome of this CEA is expressed as a ratio of incremental costs relative to incremental effects of the intervention: the incremental cost-effectiveness ratio (ICER), expressed in euros per QALY (Quality Adjusted Life Years) [14]. We performed our assessment using a dynamic model for the entire Dutch population (the RIVM Chronic Disease Model (CDM)), taking a healthcare perspective focusing on health benefits and health care costs. The health care costs included are the costs of opportunistic screening, costs of brief intervention, the costs of alcohol related diseases and costs of diseases unrelated to alcohol in life years gained. There are previous studies which investigated the cost-effectiveness of interventions similar to SBI [15], [16], [17], [18], [19] e.g. Fleming et al. 2000 [19] calculated from the societal perspective that the benefit-cost ratio of the brief intervention was 5.6:1, or $56,263 in total benefit for every $10,000 invested. Only one of the previous studies [15] investigated the cost-effectiveness of SBI in costs per QALY gained. However, this study did not take into account the medical costs of diseases unrelated to alcohol in life years gained and did not account for the fact that quality of life decreases at older ages.

## Results

The total Dutch population aged 20–65 years accounts for about 10 million people. On average 6,176,000 of them are screened during the opportunistic screening programme. As table 1 shows, 1,386,000 excessive and dangerous drinkers exist in the Dutch population aged 20–65. On average 853,000 of them are found with the screening instrument, and 577,000 of them receive the brief intervention. This results in 39,000 people becoming moderate drinkers or abstain from alcohol, which is about 3% of the amount of excessive drinkers in the Dutch population aged 20–65.

**Table 1:** Impact of the SBI. Estimates and their 95% confidence interval (between brackets).

	N
Total Dutch population aged 20–65 years	10,029,000*
Number of excessive and dangerous drinkers in total Dutch population aged 20–65 years	1,386,000*
Number of persons screened in the opportunistic screening programme in the GP practice	6,176,000 (5,740,000–6,545,000)
Number of excessive and dangerous drinkers found during the screening programme	853,000 (793,000–904,000)
Number of excessive and dangerous drinkers receiving the brief intervention	577,000 (389,000–734,000)
Number of drinkers who became moderate drinkers or abstained from alcohol	39,000 (2,000–92,000)

* Derived from the annual General Public Health and Lifestyle Survey (Dutch initials: POLS) conducted by Statistics Netherlands.

**Figure 1:**
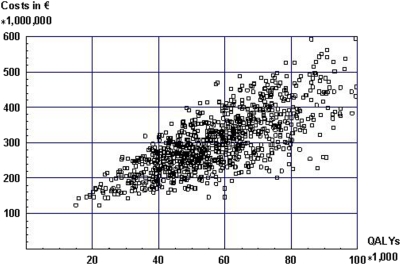
Incremental costs and effects of SBI compared to current practice scenario for the target population.

In the SBI scenario the reduction in alcohol consumption results in a decrease in the incidence of alcohol related diseases, and in a decrease in health care costs of these diseases. As a consequence, this causes a gain in life years and QALYs compared to current practice scenario (no provision of SBI). Figure 1 displays a costs (differences in intervention+lifetime health care costs) and effects (QALYs gained) plane for SBI scenario compared to current practice scenario, for different values of the model input parameters.

This figure displays the results of a PSA. Each point represents incremental costs and effects of one run of the CDM, taking as input a random sample drawn from the distributions of the model parameters. The spread of the cloud of points in the figure shows the uncertainty in the estimation of the joint distribution of costs and effects. As can be seen from this figure, health care costs increase as the amount of QALYs gained increases. If the numbers of person receiving SBI increases, both costs of SBI will increase, and the amount of QALYs gained.

Incremental life years gained, QALYs gained, health care costs incurred and the resulting ICER for the SBI scenario are shown in Table 2.

**Table 2:** Estimates of total incremental costs and effects due to SBI intervention and their 95% confidence interval (between brackets).

	SBI scenario vs. current practice scenario
Life years gained^a^ (*1,000)	82 (35/140)
QALYs gained^a^ (*1,000)	56 (24/94)
Costs SBI (* €1,000,000)^b^	61 (48/70)
Total costs differences (* €1,000,000)^b^	298 (146/514)
€ per life year gained^c^ *	700
€ per QALY gained^c^ *	1,100
€ per life year gained^c^	3,600
€ per QALY gained^c^	5,400

a Discounted with 1.5%.

b Discounted with 4%.

c QALYs and life years gained discounted with 1.5% and costs discounted with 4%.

* Only SBI costs included.

Mean incremental costs per QALY are €5,400 for the SBI scenario compared to the current practice scenario. Mean incremental costs per life year gained are €3,600 for the SBI scenario compared to the current practice scenario. When only cost of SBI itself would be included in the denominator of the ICER, mean costs per QALY and per life year gained would be respectively €1,100 and €700,- for the SBI scenario compared to current practice scenario. Costs per QALY are higher than costs per life year gained in this scenario. This is because the number of QALYs gained falls short of the numbers of life years gained, since not all life years gained are lived in perfect health.

**Figure 2:**
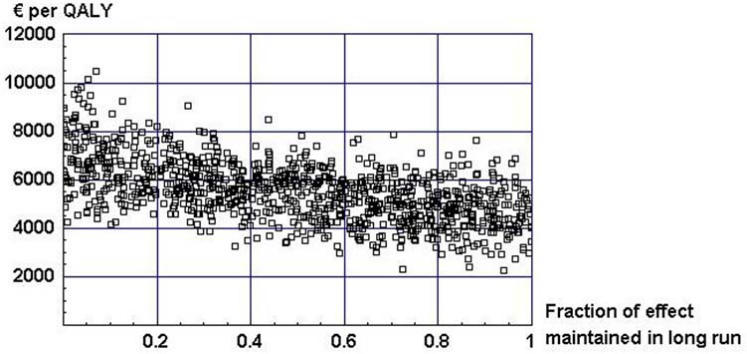
Costs per QALY gained for different fractions of effect maintained in long run.

Regarding the effect of the alcohol SBI in the long run, Figure 2 displays costs per QALY gained for different fractions of effect maintained in long run.

The figure shows that costs per QALY decrease as the fraction of effect maintained in the long run increases. This is due to an increase in the amount of QALYs gained from a maintained reduction in alcohol consumption in the long run. It should be noted that if no effect is maintained at all in the long run, the costs per QALY gained would be infinitely high.

**Figure 3:**
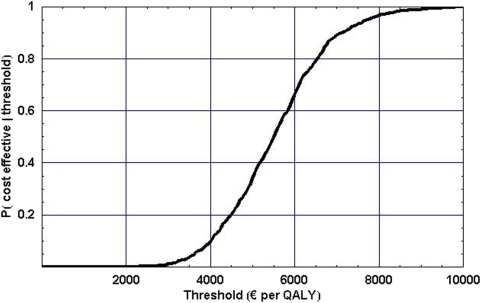
Cost-effectiveness acceptability curve for alcohol SBI scenario.

Figure 3 represents the Cost-Effectiveness Acceptability Curve for the SBI scenario, which displays the probability that the alcohol SBI is cost effective for different values of the threshold, i.e. for different monetary values placed on a QALY [14], [20].

When, for instance, a QALY is valued at €5,000, implementing alcohol SBI is cost effective with a probability of 0.4. Alcohol SBI has a probability of almost one when the society is willing to pay €10,000 per QALY, which is well below the threshold usually placed on a QALY in the Netherlands (€20,000 per QALY gained). This means that alcohol SBI can be considered cost effective.

## Discussion

Excessive alcohol use increases risks of many disorders, with associated losses of life-years and quality of life. Successful prevention of excessive alcohol consumption will therefore result in increased life expectancy and decreased health care costs. From a health care perspective, implementing SBI in primary care setting in The Netherlands would lead to health gains at a low cost. The cost-effectiveness of SBI was estimated at €5,400 per QALY gained and, thus, can be considered cost-effective.

### Effectiveness

In several randomized studies, alcohol brief intervention has been proven to be effective in reducing excessive alcohol use [7], [8], [9], [10], [11]. However, Beich et al. [11] indicate that the numbers of patients needed to screen (NNS) in general practices is quite high to achieve benefits, because only two to three patients per thousand screened benefit from the laborious activities entailed in screening. But, we demonstrated that opportunistic screening is relatively cheap since all patients are already present in the waiting room of the GP.

### Cost-effectiveness

Previous research studies investigating the cost-effectiveness of interventions similar to alcohol SBI also considered this type of intervention to be cost-effective [15], [16], [17], [18], [19]. However, these studies did not always express the outcome measure in terms of QALYs. Only Solberg et al. [15], when ranking the health impact and cost effectiveness of alcohol primary care intervention to reduce alcohol misuse did this in terms of QALYs gained. However, Solberg et al. [15] did not take into account the medical costs of diseases unrelated to alcohol in life years gained. Furthermore, they did not take into account the fact that quality of life decreases at older ages [21]. Wutzke et al. [16] expressed the outcome measure only as costs per life year saved by preventing alcohol-related deaths, and did not include the initial costs of screening. Babor et al. [17] took as their outcome measure the intervention costs per reduction in alcohol consumption per patient, again not the amount of QALYs gained.

### Assumptions

In this study, we made some assumptions that need to be confirmed by further research. The long term effects of alcohol SBI are debatable. Babor et al. [22] indicated that long term effects on population health had not yet been demonstrated. Wutzke et al. [23] conclude that there is no effect of alcohol SBI without any regular check-ups after 10 years. However, Nilssen [24] conclude that the impact of brief interventions for individuals who are at increased risk but not (yet) meeting the DSM-IV criteria for alcohol dependency appears to be long lasting, up till 9 years after the provision of alcohol SBI. In our study, we took into account the uncertainty regarding the long term persistence of the effect by incorporating a “bandwidth” for the part of the short term decrease in alcohol consumption that is conserved in the long run, including the possibility that no effect remains. It is obvious to note that if there would be no more effect of alcohol SBI in the long term, then the cost-effectiveness ratios of this intervention would increase substantially.

### Limitations

One limitation of our analysis is that external effects of implementing the alcohol SBI were ignored because a health care perspective was taken. We have solely focused on health care costs, ignoring broader costs and consequences (like damage done due to violence and accidents induced by drinking) of a reduction of excessive alcohol consumption which fall outside the scope of the health care budget. However, these broader costs seem to be related mostly to drinking patterns [25]. The model we employed only models the relation between average alcohol consumption on quality of life, mortality and health care costs which corresponds well to the outcome measures reported in the brief intervention trials. In case brief intervention would have any effect on alcohol patterns, brief intervention is expected to be more cost-effective, and maybe even cost-saving.

In summary, prevention of excessive alcohol use by implementing the alcohol SBI in a GP setting appears to be cost-effective, with mean incremental costs of €5,400 per QALY gained. The findings of this alcohol SBI economic evaluation implicate that such an intervention is a wise use of health care resources to reduce the public burden of diseases related to excessive alcohol use.

## Methods

### Intervention

SBI for excessive alcohol use in medical settings entails two elements: *1)* opportunistic screening in a primary care setting (i.e. a General Practice) to identify excessive drinkers; *2)* brief intervention: a low-intensity, short-duration counselling intervention of 10 to 15 minutes, with feedback about drinking, advice and goal setting, and a follow-up contact (one or more discussions lasting 10 to 15 minutes with a primary care physician).

### Scenarios

To estimate the effects of SBI, the two following scenarios were compared:

- **Current Practice scenario**: in this scenario nobody in the Dutch population is screened for alcohol consumption and alcohol consumption patterns remain at their current level;
- **SBI scenario**: all persons between age 20 and age 65 who visit the GP within one year are screened and those who are identified as excessive drinkers receive the brief intervention. Due to brief intervention alcohol consumption patterns are altered.

The SBI scenario entails the following steps [26]:

1. All patients visiting general practice are approached by a GP-assistant when they are in the waiting room during a regular visit, i.e. not specifically for alcohol related complaints. They are asked to complete the AUDIT-questionnaire, a well-validated alcohol screening instrument developed by the World Health Organization [27];
2. Completed questionnaires are scored by the GP. Those identified as excessive drinkers (an AUDIT-score of 8+) and not meeting the DSM-IV criteria for alcohol dependency are administered the Brief Intervention;
3. The GP provides follow-up sessions at 6 and 12 months.

### The RIVM Chronic Disease Model

We estimated incremental health effects and costs by comparing the two scenarios. To extrapolate from decreased alcohol consumption as a result of the intervention to effects on health care costs, life years gained and QALYS gained, we used the RIVM Chronic Disease Model (CDM) [28], [29], [30]. The CDM is a tool that relates (changing) prevalences of risk factors in the general population, such as smoking, overweight and alcohol consumption, to the occurrence of chronic diseases. It has been used for projections into the future, as well as to estimate health adjusted life expectancy and to perform cost effectiveness analysis [21], [31], [32], [33], [34]. The model describes the life course of cohorts in terms of changes between risk factor classes and changes between disease states over time. It allows for co-morbidity and includes epidemiological data on the most important chronic diseases and their risk factors. Risk factors and diseases are linked through relative risks for disease incidence. Alcohol consumption is included as a risk factor that is linked to the following diseases: coronary heart disease, stroke, oesophagus cancer, breast cancer, oral cavity cancer and larynx cancer. Alcohol consumption is divided into the following classes: abstinence (no alcohol consumption), moderate alcohol consumption (less than 2 standard drink units per day, for women, or 4 units for men), excessive alcohol consumption (between 2 and 4 standard drink units per day, respectively 4 and 6 for men) and dangerous alcohol consumption (more than 4, respectively 6 standard drink units per day) [35]. The distribution over these classes of alcohol consumption patterns of the current Dutch population was estimated using data from the annual General Public Health and Lifestyle Survey (Dutch initials: POLS) conducted by Statistics Netherlands [36].

The relative risks for the diseases related to alcohol consumption and all cause mortality employed in the CDM were all derived from the meta-analysis by Holman et al. [35]. This was the only study that included relative risk estimates for the alcohol categories employed in the CDM for both diseases and mortality, and that provided estimates of all-cause mortality. Such a category of all-cause mortality was not used in other studies on relative risks of alcohol consumption. In the simulation model we used these estimates of relative risks on total mortality to estimate the effects of alcohol on mortality through causes of death that are not explicitly in our model.

In essence, our model compared the current distribution of alcohol consumption according to the classes distinguished in the model to the distribution that would result from the intervention. In order to estimate this new “post-intervention” distribution, we again used the POLS data mentioned above, but this time subtracting from the raw data the average decrease in alcohol consumption due to SBI for every individual in the data set having the characteristics of the target population, and then re-estimating the alcohol consumption distribution.

To calculate the decrease in alcohol consumption due to SBI we multiplied the sensitivity of the screening instrument by the decrease in alcohol consumption times the long term maintenance fraction. The long term maintenance fraction is the fraction of the decrease in alcohol consumption that can be sustained in the long run. It is assumed that only this fraction results in health gains.

To compute health effects in terms of QALYs, the CDM couples disability weights from the Dutch Burden of Disease Study to disease prevalence rates [28]. For diseases causally related to alcohol consumption, we used the CDM to estimate diseases prevalence rates as a function of time. To capture the impact on quality of life of diseases not related to alcohol consumption during life years gained we used age and gender specific prevalence rates as reported in the Dutch Burden of Disease Study [37]. Cost of illness (COI) data from the Netherlands for the year 2003 [38] served to estimate health care expenditure conditional on disease status and age [31], [32]. Annual disease costs per patient were multiplied by the projected future prevalence numbers for each alcohol related chronic disease in the model. Costs of all other diseases, incurred during life years gained, were calculated as the product of the numbers of “survivors” and the category of ‘remaining costs’. These latter equal the difference between total health care costs and the costs of the alcohol related diseases incorporated in the model. They include, for instance, the costs of mental and behavioural disorders.

### Discounting

To calculate cost-effectiveness ratios, yearly differences in model outcomes between intervention and current practice scenario were discounted and added over the time horizon to find net present values for incremental life years gained, QALYs gained, and health care costs. Future costs and effects were discounted at the annual percentages of 4% for costs and 1.5% for effects which are recommended in the Dutch guidelines [39]. The time horizon chosen was 100 years since by then the cohorts that experienced the intervention will have become extinct (that is, the cohort was “followed to extinction”). We used consumer price indices to adjust all cost to a 2008 price level.

### Derivation of the input parameters for the RIVM CDM

To quantify the costs and effects of the SBI, we needed to make some assumptions and determine several parameters. Here we describe how we determined the parameters which we used as input for the RIVM CDM.

### The effectiveness of the Screening and Brief Intervention

First, to estimate the numbers of patients willing to undergo screening (screening uptake) we calculated a pooled estimate from all studies described in Solberg et al. [15], [40], [41], [42]. We fitted a logistic random effects regression model with only a constant to obtain the estimate and the uncertainty surrounding it. Mean screening uptake was 86%. Second, for the sensitivity and specificity of the screening instrument, employing the same methodology we calculated a pooled estimate from all studies described in Fiellin et al. [12]. They analyzed the AUDIT questionnaire as the screening instrument with a cut-off score of 8+, which indicates that a person drinks excessively. Mean estimate for the sensitivity of the screening instrument was 69%; and for specificity 94%. Third, as an estimate of the decrease in alcohol consumption as a result of the brief intervention, we took the values provided by Bertholet et al. [10] in their systematic review, namely on average a reduction in weekly ethanol intake of 38 gram per person. With regard to the persistence of the effects of SBI in the long run, three studies were found showing contrasting results. Babor et al. [22] indicated that brief intervention can reduce alcohol use for at least 12 months in non-dependent heavy drinkers, but long term effects on population health had not yet been demonstrated. Wutzke et al. [23] concluded that there is no remaining effect after a period of 10 years when there are no regular check-ups. However, Nilssen [24] concluded that the impact of a brief intervention appears to be long lasting, up till 9 years after the provision of the intervention.

In our study, we assumed that health effects only accrue if behavioural changes are maintained lifelong. The fraction of the initial decrease in alcohol consumption that is maintained in the long run was drawn from a uniform distribution with minimum 0 and maximum 1.

### Costs of the SBI intervention

The costs of opportunistic screening and subsequent intervention were calculated by using a so-called bottom-up method as advocated in the Dutch Pharmacoeconomic guidelines [43]. All elements of the screening and intervention were identified and thereby the resources needed. These units and unit prices are displayed in Table 3.

**Table 3:** Costs of the SBI intervention, per person.

Type of costs	Unit	Unit price	Costs
Approaching patients by GP-assistant	1 min	0.66	€0.66
Checking AUDIT score 8+, by GP	1 min	2.19	€2.19
Further screening by GP	5 min	2.19	€10.95
Brief intervention by GP	10 min	2.19	€21.9
Follow-up sessions by GP	20 min	2.19	€43.8

Altogether, the screening process costs about €14,- per person. Providing brief intervention costs about €22,- per person, and follow-ups costs are €44,- per person.

### Target population

In this modelling study, the target population consisted of all people between age 20 and 65 who visit the GP in a particular year, which according to Statistics Netherlands (CBS), is approximately 7.2 million [6].

### Sensitivity analysis

We used the technique of Probabilistic Sensitivity Analysis (PSA) to account for uncertainty in the model input parameters. PSA lets uncertainty in the input parameters be reflected in the model output (the ICER). We identified and addressed the following sources of uncertainty in our base-case ICER estimate: the uncertainty around the relative risk values [35], the values of screening uptake [15], the sensitivity and specificity of the screening instrument [12], the follow-up rate of the brief intervention [10], decrease in alcohol consumption [10], and, the fraction of decrease in alcohol consumption maintained in the long run [22], [23], [24]. Table 4 summarizes the assumptions and the distributions for the input parameters used in the different scenarios. In order to perform the analysis the model was run 1000 times. For each run of the model, at random a number is drawn for each of the “uncertainty” parameters from the distribution specified for that parameter (see table 4). This leads to a different output of the model for each run. The variability in outcome is then a reflection of the uncertainty due to the input parameters.

**Table 4:** Summary of assumptions and input data.

	Current practice scenario	SBI scenario
**Assumptions**		
*Discount rate*	4% costs and 1.5% effects	4% costs and 1.5% effects
*Time horizon*	80 years (lifetime)	80 years (lifetime)
*Target population*	Risky drinkers aged between 20 and 65 who visit the GP yearly (50%)	Risky drinkers aged between 20 and 65 who visit the GP yearly (50%)
**Distributions used in PSA**		
*Relative risks*	Lognormal distributions derived from meta analyses Holman [35]	Lognormal distributions derived from meta analyses Holman [35]
*Fraction of the target population that agrees to be screened*		Logit distribution (1/(1+e^−x^) with x normally distributed^a^:
		Mean: 1.802
		SD: 0.237
*Sensitivity of the screening instrument*		Logit distribution (1/(1+e^−x^) with x normally distributed^b^:
		Mean: 0.800
		SD: 0.490
*Specificity of the screening instrument*		Logit distribution (1/(1+e^−x^) with x normally distributed^b^:
		Mean: 2.763
		SD: 0.168
*Fraction for second interview*		Logit distribution (1/(1+e^−x^) with x normally distributed^a^:
		Mean: 1.149
		SD: 0.216
*Decrease in alcohol consumption*		Normal distribution^c^
		Mean: 1.149
		SD: 0.216
*Fraction of decrease in alcohol consumption maintained*		Uniform distribution
		Minimum: 0
		Maximum: 1

a Pooled estimate from all studies selected and described in Bertholet et al. [10]. A logistic random effects regression analyses with only a constant was carried to obtain the effect estimate and the uncertainty surrounding it.

b Pooled estimate from all studies selected and described in the systematic review by Fiellin et al. [12]. A logistic random effects regression analyses with only a constant was carried to obtain the effect estimate and the uncertainty surrounding it.

c Bertholet et al. [10].
